# A Review on (Hydro)Porphyrin-Loaded Polymer Micelles: Interesting and Valuable Platforms for Enhanced Cancer Nanotheranostics

**DOI:** 10.3390/pharmaceutics11020081

**Published:** 2019-02-15

**Authors:** Bruno F. O. Nascimento, Nelson A. M. Pereira, Artur J. M. Valente, Teresa M. V. D. Pinho e Melo, Marta Pineiro

**Affiliations:** CQC and Department of Chemistry, University of Coimbra, Rua Larga, 3004-535 Coimbra, Portugal; nascimento@ci.uc.pt (B.F.O.N.); npereira@qui.uc.pt (N.A.M.P.); avalente@ci.uc.pt (A.J.M.V.); tmelo@ci.uc.pt (T.M.V.D.P.e.M.)

**Keywords:** nanotheranostics, photodynamic therapy, porphyrin, micelles, nanoparticles

## Abstract

Porphyrins are known therapeutic agents for photodynamic therapy of cancer and also imaging agents for NIR fluorescence imaging, MRI, or PET. A combination of interesting features makes tetrapyrrolic macrocycles suitable for use as theranostic agents whose full potential can be achieved using nanocarriers. This review provides an overview on nanotheranostic agents based on polymeric micelles and porphyrins developed so far.

## 1. Introduction

Cancer remains a great threat to human health worldwide. Early detection, accurate diagnosis, and effective treatment are the keys to increasing cancer survival rates. Innovative solutions for the poor selectivity and sensitivity of conventional diagnosis techniques, systemic toxicity, and low selectivity of cancer drugs and drug resistance, among other problems, will increase the outcome of cancer therapies [1,2,3].

Theranostic systems combining imaging and therapy can synergistically act to improve all stages of patient care, from screening and diagnosis to therapeutic decisions, as well as treatment itself and treatment follow-up [4,5,6,7,8].

The physical and chemical properties of nanoparticles (NPs), such as small size or high surface-to-volume ratio, are appropriate for achieving efficient cell uptake and high loading. Facile surface functionalization opens the possibility to append targeting moieties and to encapsulate individual entities together in the same environment, minimizing cellular immunostimulation and toxicity. The ability to increase target-to-background contrast for imaging applications makes them attractive candidates for theranostic applications [9,10].

Nanotheranostics embracing multiple techniques are expected to arrive to comprehensive diagnosis, molecular imaging, and individualized treatment, providing high-quality cancer imaging and enhancing the therapeutic effect. Theranostic nanotechnologies may provide patients with personalized treatment resulting in better prognoses [11]. The ideal theranostic probe needs to have the following characteristics: be able to detect the tumor, high signal-to-noise ratio allowing high sensitivity, high specificity to the target, and be non-toxic at the concentration needed for detection and therapeutic effect [12,13].

Porphyrins and related macrocycles have unique properties that have made them the focus of attention in diverse diagnosis imaging applications and also in cancer therapies. Porphyrins can be used in fluorescence imaging, magnetic resonance imaging (MRI), photodynamic therapy (PDT), sonodynamic therapy (SDT), boron neutron capture therapy (BNCT), and radiation therapy. In fact, porphyrins have been widely explored for theranostics [14,15,16,17,18,19,20,21,22,23,24,25].

Most of the porphyrins designed as therapeutic agents are hydrophobic, with poor solubility in water. Aggregation in aqueous systems is a common problem [26,27] which has been the driving force behind the search of efficient delivery systems [28,29,30], including nanoparticles. 

Micelles, vesicles, and polymer conjugates are the most commonly used delivery vectors of organic origin [31,32]. Micelles are self-assembling colloidal structures comprising, in an aqueous solution, a hydrophobic core and a hydrophilic shell, with narrow size distribution and sizes usually below 100 nm that have been emerging as multifunctional nanotherapeutic platforms for cancer imaging and delivery of chemotherapeutics applied at different stages of clinical trials [33,34]. The use of micellar nanostructures and porphyrins or hydroporphyrins, as theranostic agents, is the focus of the present review.

## 2. Micellar Nanostructures for Nanotheranostics Based on PDT and Imaging

Elbayoumi et al. reported the development of liposomes and lipid-core micelles made of poly(ethylene glycol)-phosphatidylethanolamine (PEG-PE) conjugates, and immunomicelles through modification with nucleosome-specific monoclonal antinuclear autoantibody 2C5 (mAb 2C5), which specifically recognizes cell surface-bound nucleosomes in several cancer cells [35]. The efficacy of PEG-PE micelles, and tumor-targeted immunomicelles as delivery systems for *meso*-tetraphenylporphyrin (TPP) and the photosensitizing action were investigated, in vitro, using MCF-7 human breast adenocarcinoma cell lines. TPP-loaded PEG-PE micelles (plain or immuno) were essentially non-toxic on their own, with no dark toxicity and around 95% of cell viability at maximum TPP concentration (50 μg/mL) and incubation time (18 h). Nonetheless, a strong increase in cytotoxicity was observed for TPP-containing PEG-PE micelles and immunomicelles compared to TPP alone, after light irradiation. At both maximum TPP concentration and pre-irradiation incubation time, the usage of TPP-loaded PEG-PE micelles yielded ca. 65% cancer cell death; on the other hand, the use of TPP in 2C5 immunomicelles resulted in the demise of 95% of cancer cells. This could be related to the improved binding of mAb 2C5-modified immunomicelles to tumor cells.

Fluorescence microscopy imaging studies of PDT-treated human Lewis lung carcinoma (LLC) cells were performed to confirm that the photocytotoxicity observed was due to an increase of apoptosis, the main mechanism of PDT-associated cell death. The cells treated with TPP-loaded mAb 2C5 immunomicelles clearly exhibited more DNA fragments when compared to TPP-containing plain micelles; apoptotic DNA fragments could hardly be seen in the dark and irradiated control groups. This noticeable rise in the TPP-induced cancer cell-killing ability of these immunomicelles may be explained by the enhanced specific binding and, most likely, micelle internalization in cancer cells, which allowed for the accumulation of the photosensitizer and, consequently, a more potent photodynamic effect.

**Figure 1:**
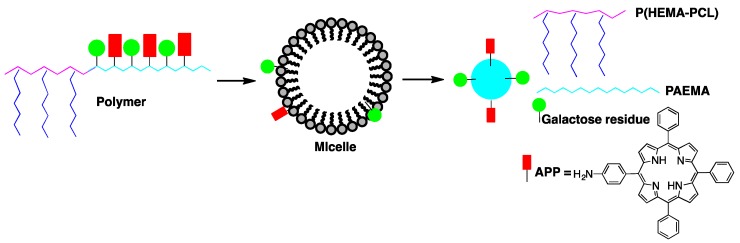
Schematic illustration of the core–shell micelle self-assembled from Gal-APP-PAEMA-PCL.

The targeting and PDT efficiency of amphiphilic galactosyl (Gal) and 5-(4-aminophenyl)-10,15,20-triphenylporphyrin (APP) poly(2-aminoethyl methacrylate)-polycaprolactone (Gal-APP-PAEMA-PCL) micelles was assessed by Wu et al. [36]. Poly(2-aminoethyl methacrylate)-polycaprolactone (PAEMA-PCL) was prepared by a combination of ring opening polymerization and reversible addition–fragmentation chain transfer (RAFT) polymerization, with Gal-APP-PAEMA-PCL being obtained after conjugation with lactobionic acid and mono-aminoporphyrin (APP), Figure 1.

Fluorescence emission spectroscopy data showed that for both APP-PAEMA-PCL and Gal-APP-PAEMA-PCL micelles there are two APP molecules per 100 AEMA units in the polymer chain. The size of the micellar vehicles was estimated to be less than 50 nm as seen by transmission electron microscopy (TEM); however, by dynamic light scattering (DLS), the mean sizes for APP-PAEMA-PCL and Gal-APP-PAEMA-PCL micelles were 60 and 68 nm, respectively. This difference was justified by the fact that DLS measures the hydrodynamic diameter of micelles in aqueous solution, whereas TEM determines the diameter of the freeze-dried micelles. The encapsulation efficiency and drug loading values of anticancer drug doxorubicin (DOX) by APP-PAEMA-PCL and Gal-APP-PAEMA-PCL micelles were calculated as 15.6% and 18.2%, and 13.2% and 16.5%, respectively. The in vitro DOX release profiles of the two types of carriers differ slightly, with the cumulative release of Gal-APP-PAEMAPCL micelles being faster than that of APP-PAEMA-PCL micelles. The calculated fluorescence quantum yields (Φ_F_) increased in the order of APP-PAEMA < APP-PAEMA-PCL < Gal-APP-PAEMA-PCL, which the authors ascribed to PAEMA and PAEMA-PCL segments conjugated to the sensitizer. This can, in turn, prevent porphyrin molecules from overlapping, thus avoiding fluorescence quenching and increasing the fluorescence quantum yields. The in vitro cytotoxicity (dark toxicity) was evaluated utilizing human hepatocellular liver carcinoma (HepG2) and human laryngeal carcinoma (HEp2) cells, and no significant decrease in cell viability was detected in any case. However, all porphyrin-incorporated micelles demonstrated cytotoxicity against HepG2 and HEp2 after light irradiation. Half maximal inhibitory concentration (IC_50_) values of 0.2 and 0.5 mg/mL for HepG2 cells, and 0.4 and 0.5 mg/mL for HEp2 cells were determined for Gal-APP-PAEMA-PCL and APP-PAEMA-PCL, respectively.

The increase of phototoxicity observed using micelles containing galactosyl hepatic-targeting moieties could be rationalized by an increase of internalization due to the presence of targeting groups in the case of HepG2 cells. In fact, fluorescence microscopy studies showed that the favored sites of intracellular localization were the lysosomes of HepG2 and HEp2 cells for both micelles, and a higher accumulation of Gal-APP-PAEMA-PCL in HepG2 cell lines when comparing with Gal-APP-PAEMA-PCL in HEp2 cells, while the accumulation of APP-PAEMA-PCL is independent of the cell type. 

The preferential intracellular localization in lysosomes of both HepG2 and HEp2 cells might suggest a pH-responsive behavior of the nanocarriers, due to the existence of amino groups in PAEMA segments, which can play a significant role in determining the preferential localization sites of the micelles within cells.

**Figure 2:**
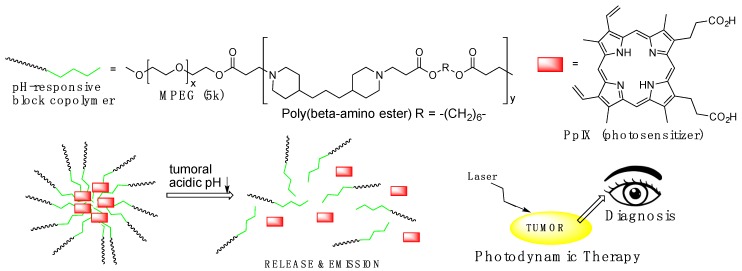
Schematic illustration of the micellization and demicellization processes of protoporphyrin IX (PpIX)-pH-responsive polymeric micelles (pH-PMs) and simultaneous tumor photodiagnosis and photodynamic therapy in vivo.

The difference of pH values observed in tumor tissue, usually around 6.4–6.8, and normal tissue (pH = 7.4), has been used as the base for the development of more efficient therapeutic approaches in drug delivery and biomedical imaging. pH-responsive block copolymer (Pluronic) micelles, capable of demicellization at the slightly acidic pH of tumor tissue, were used as delivery systems, increasing the accumulation in tumor tissues and releasing the sensitizer to produce fluorescence and reactive oxygen species (ROS) for simultaneous diagnosis and therapy purposes in the tumor site, Figure 2. Koo et al. combined hydrophilic methoxypoly(ethylene glycol) (MPEG) with pH-sensitive poly(β-amino ester), which yielded a pH-responsive MPEG-poly(β-amino ester) block copolymer due to its tertiary amines (pK_b_ = 6.5), with an average molecular weight of 17.4 kDa. Loading of hydrophobic protoporphyrin IX (PpIX) into polymeric micelles of MPEG-poly(β-amino ester) block copolymer gave PpIX-loaded pH-responsive polymeric micelles (PpIX-pH-PMs) with an average size of about 122 nm, which was far larger than that of the unloaded micelles (42 nm), an indication that the PpIX molecules were, in fact, trapped in the hydrophobic inner cores of the micellar platforms [37].

The intracellular localization of free PpIX and PpIX-pH-PMs was investigated by confocal microscopy using SCC7 human squamous cell carcinoma cell lines. The uptake of PpIX remained equal at both pH 6.4 and 7.4. However, while PpIX-pH-PMs was detected through a weak fluorescence signal at the cell membranes at physiological pH (i.e., 6.4), a strong fluorescence signal of PpIX was observed in the cytoplasm. This pH-dependent cellular uptake of PpIX was explained as derived from the rapid demicellization of PpIX-pH-PMs under weakly acidic conditions, leading to the quick discharge of PpIX from the polymeric micelles and subsequent internalization into cells. 

**Figure 3:**
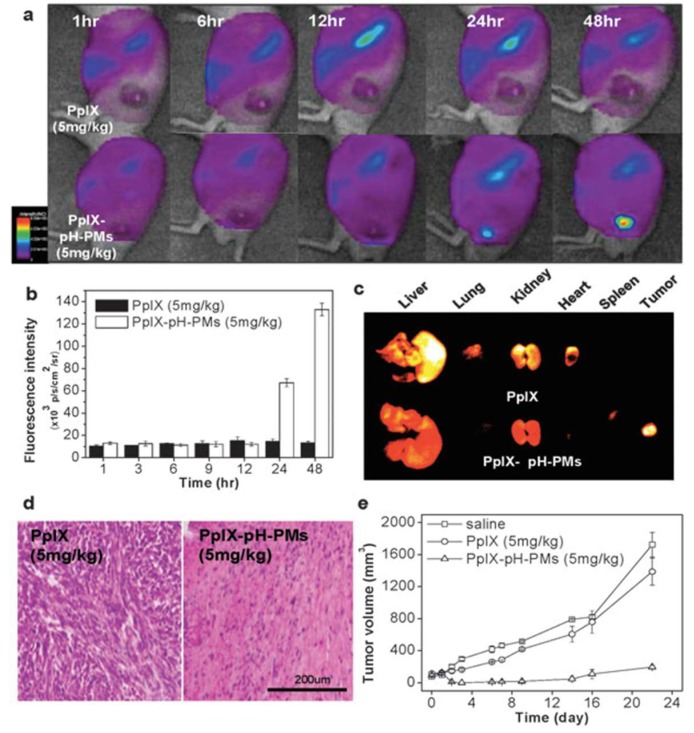
Non-invasive fluorescence imaging and photodynamic therapy (PDT) with PpIX-pH-PMs in live SCC7 tumor-bearing mice. In vivo time-dependent whole body imaging after injection (**a**); in vivo quantification of tumor target specificity of free PpIX and PpIX-pH-PMs (**b**); ex vivo images of organs (liver, lung, spleen, kidney, heart) and tumors (**c**). (**d**) H&E staining of tumor tissues 10 days after treatment. (**e**) Tumor growth of SCC7 tumor-bearing mice treated with drug injection and irradiation. Results represent means ± SDs. (*n* = 2). Reproduced from Reference [37] with permission from the Royal Society of Chemistry, 2010.

PpIX-pH-PMs show no cytotoxicity in the dark; however, a pronounced photocytotoxicity was observed in vitro, with both free PpIX and PpIX-pH-PMs at pH = 6.4. The tumor specificity of free PpIX and PpIX-pH-PMs in live SCC7 tumor-bearing mice was also evaluated. Using free PpIX, very weak fluorescence signals were observed in tumors, making it difficult to distinguish tumor from normal tissues, while strong fluorescence signals were mostly observed in the liver. In the case of PpIX-pH-PMs, very strong fluorescence intensity at the tumors was detected 24 h after injection; moreover, the tumors could be easily delineated from the neighboring background tissue (Figure 3a). Additionally, the fluorescence intensity in tumor tissue 48 h after PpIX-pH-PM injection were 10-fold higher than that of free PpIX, Figure 3b. Upon ex vivo imaging of excised organs (liver, lung, spleen, kidney, heart) and tumors, Figure 3c, PpIX-pH-PM-treated mice exhibited the strongest fluorescence intensity in the tumors, and PpIX was undetectable in other normal organs except in the liver and kidney, where PpIX was promptly metabolized.

**Figure 4:**
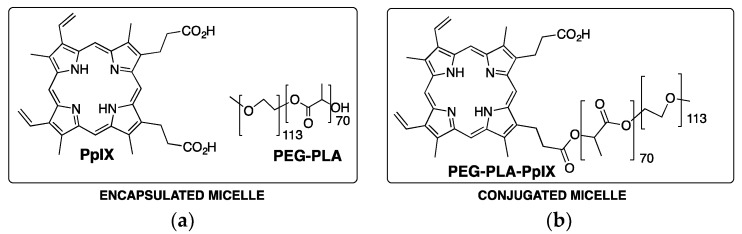
PEG-PLA and photosensitizer (**a**) and PEG-PLA-photosensitizer (**b**) structures used for the preparation of encapsulated and conjugated micelles, respectively.

Biocompatible and biodegradable block copolymer, poly(ethylene glycol)-*b*-poly(d,l-lactide) (PEG-PLA) was used to produce micellar nanocarriers for the delivery of PpIX [38]. Two types of PEG-PLA-PpIX micelles were produced: one through a non-covalent hydrophobic encapsulation methodology and another by covalent bond of PpIX to the hydroxyl group of PEG-PLA, Figure 4. TEM characterization shows that PpIX-containing micelles had a spherical morphology, and DLS analysis indicated that the micelle diameters were around 30 nm. Depending on the incorporation strategy (physical encapsulation vs. covalent conjugation) and loading density, PpIX may occur as a monomer, dimer, or aggregate inside the micelle core, which directly affect the fluorescence intensity and ROS production and, therefore, the therapeutic efficiency of the resulting micelles in aqueous solution.

Micelles with lower PpIX loading density (e.g., 0.2%) exhibit brighter fluorescence and higher singlet oxygen yield than those with higher PpIX loading density (e.g., 4%). Interestingly, in vitro PDT studies carried out with H2009 human lung cancer cell lines showed a contrasting trend. Particularly, 4% PpIX-conjugated micelles demonstrated the best PDT index, with the highest photocytotoxicity and relatively low dark toxicity. Confocal microscopy was employed by the authors to examine the micelle uptake and PpIX release in H2009 cells. At 4% drug loading, both micelle formulations had very low fluorescence emissions in cell culture media due to PpIX quenching within the micelle core. With 4% PpIX-encapsulated micelles, a rapid increase in fluorescence was observed in the first 4 h, which was assigned to the release of free PpIX from intact micelles, as well as to dissociation of micelles into PEG-PLA unimers and free PpIX. In the case of 4% PpIX-conjugated micelles, higher intracellular fluorescence intensity was also observed over 0.2% PpIX-loaded micelles, suggesting the dissociation of some micelles into PEG-PLA-PpIX unimers. Conjugated micelles with 4% loading density were much more stable and showed slower micelle dissociation kinetics when compared with 4% encapsulated micelles in cell culture medium, which is consistent with the fast equilibrium of the latest and the steady increase of conjugated micelles in intracellular fluorescence measurements.

Micelles of pH-responsive poly(2-ethyl-2-oxazoline)-*b*-poly(d,l-lactide) (PEOz-*b*-PLA) diblock copolymer has been used as drug carrier for photodynamic therapy using *meso*-tetra(3-hydroxyphenyl)chlorin (*m*-THPC) as sensitizer. Micelles provide an appropriate environment for loading and significantly reduce the skin phototoxicity of this sensitizer, while no significant difference was observed between *m*-THPC and the *m*-THPC-containing micellar system concerning tumor accumulation and PDT efficacy [39]. 

**Figure 5:**
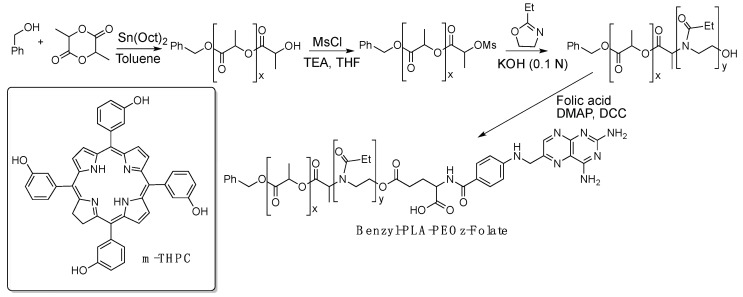
Synthetic strategy for folate-conjugated PEOz-PLA copolymer used to prepare pH-responsive micelles to encapsulate *m*-THPC.

Folate receptor, a cancer-associated protein, has been used to selectively target folate-conjugated nanocarriers and improve tumor targeting, preventing photodamage to normal tissues and increasing PDT effectiveness. To take advantage of this selective receptor, Lai and colleagues synthesized a folic acid conjugate with poly(2-ethyl-2-oxazoline)-*b*-poly(d,l-lactide) (PEOz-PLA-folate) copolymer, Figure 5 [40]. The micelles show a particle size around 100 nm and polydispersity index of 0.28. The confocal microscopy and spectrofluorometric results clearly show that micelles of PEOz-PLA-folate loaded with *m*-THPC have a loading efficiency of ca. 85%. This formulation not only decreases the skin phototoxicity, but also increases the photodynamic therapy efficiency reducing the usual PS dosage by one-third to achieve similar results in vivo. 

**Figure 6:**
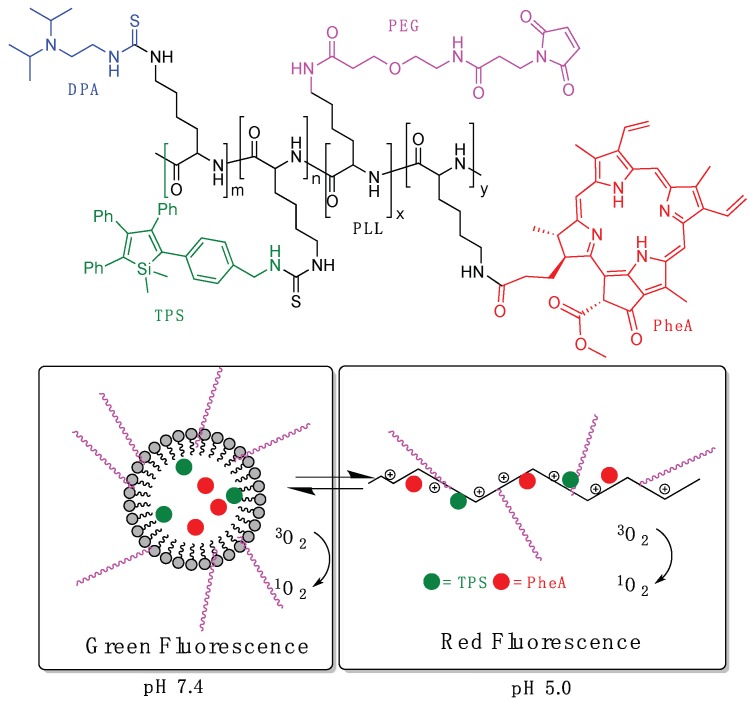
Structure of PLL-*g*-PEG/DPA/TPS/PheA (probe 1) and schematic illustration of the pH-activable probe 1.

In 2015, Tang et al. reported a pH-responsive nanoprobe for tracing cancer therapy obtained from a poly-l-lysine polymer (PLL) conjugated with a pH-responsive diisopropylamino side group (DPA, pKa = 6.1) to promote reversible assembly/disassembly, the photosensitizer pheophorbide-a (PheA), and tetraphenylsilole (TPS), showing aggregation-induced emission (AIEgen) characteristics, Figure 6 [41]. Probe 1 also incorporated a PEG block that was further conjugated with cRGD-SH to increase the targeting ability to cancer cells overexpressing α_v_β_3_ integrin. For control experiments, a non-pH-responsive polymer (PLL-*g*-PEG/Hex/TPS/PheA, probe 2) was also synthesized. The degrees of substitution in probe 1, per unit of PLL, were determined as 2.4% for TPS, 1.9% for PheA, 81.2% for DPA, and 6.3% for PEG. Probe 1 self-assembled into spherical nanoparticles in aqueous media with hydrophobic DPA/TPS/PheA core and hydrophilic PEG outer shell, presenting a hydrodynamic diameter (*R*_h_) of 115 nm at pH 7.4. At a lower pH value (5.0), the change in hydrophilicity by the protonation of DPA induced the disassembling of nanoparticles (*R*_h_ = 15.0 nm), and very few irregular aggregates were observed by TEM. As a result, at physiological conditions (pH 7.4), the self-assembled probe showed weak red fluorescence from PheA due to aggregation-caused quenching (ACQ), but presented a strong green fluorescence from TPS. On the other hand, at pH 5, the green fluorescence of TPS is decreased while the red fluorescence from PheA intensifies. They also demonstrated that the production of ROS in solution is suppressed when the probe is in micellar form at pH 7.4, and was recovered when it disaggregates at pH 5, Figure 6. Furthermore, both probe 1 and probe 2 were conjugated with cRGD-SH to yield the targeted RGD-probe 1 and RGD-probe 2, respectively. Evaluation of targeting ability of RGD-probe 1 to different cancer cells, integrin-positive MDA-MB-231 cells, or integrin-negative MCF-7 or 293T cells, confirmed that the RGD-probe 1 showed higher affinity to MDA-MB-231 cells. Under white light irradiation (0.25 W/cm^2^, 1 min), only RGD-probe 1 demonstrated substantial photocytotoxicity to human breast cancer cell line MDA-MB-231 (incubation with PS for 4 h) related with the lysosomal membrane disruption activating cell apoptosis.

**Figure 7:**
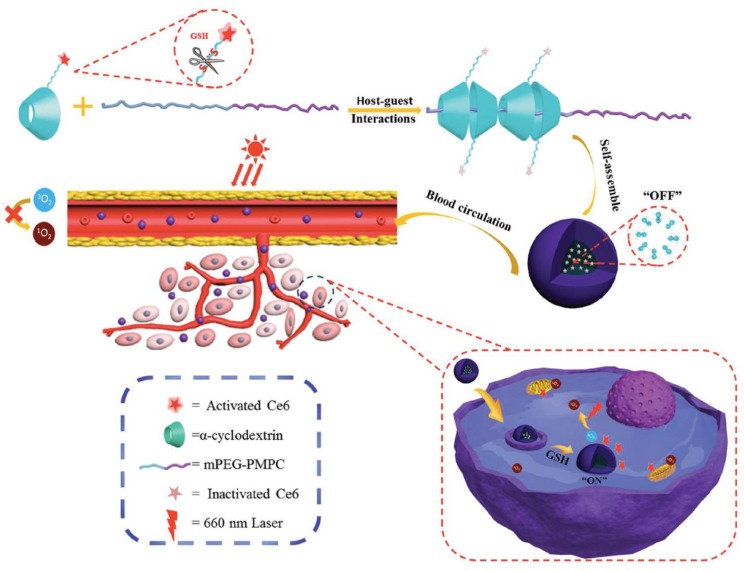
Schematic illustration of glutathione (GSH)-activable PS-conjugated pseudopolyrotaxane nanocarriers for photodynamic theranostics. Reproduced from Reference [42] with permission from *Small*, 2016.

Jin et al. linked Chlorin e6 (Ce6) to an α-cyclodextrin(α-CD)-based pseudopolyrotaxane nanocarrier through glutathione (GSH)-cleavable disulfide linkers, and PEG-*b*-poly(2-methacryl-oyloxyethyl phosphorylcholine)), developing a redox-responsive photodynamic theranostics platform (α-CD-ss-Ce6), Figure 7 [42]. TEM analysis showed that the micelles presented a spherical morphology with a uniform size of 60 nm, loading values of 20%, and diameters of ca. 106 nm, which were determined for micelles by UV–vis absorption and DLS, respectively. Both fluorescence and capability of ROS generation are highly self-quenched when α-CD-ss-Ce6 micelles are in their native state while, upon irradiation and in the presence of reducing agent dithiothreitol (DTT), they exhibited strong fluorescence and ROS production, probably due to the release of Ce6 from the micelles. 

**Figure 8:**
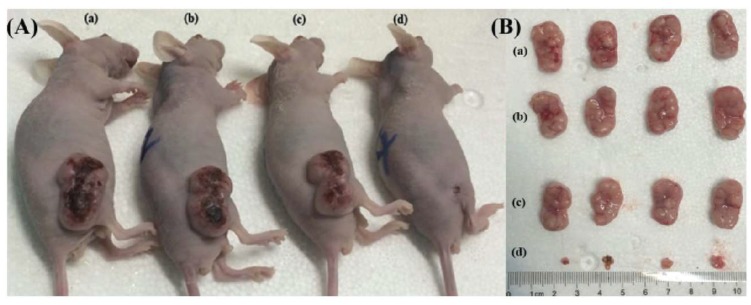
(**A**) Human oral epidermoid carcinoma (KB) tumor-bearing nude mice and (**B**) excised KB tumor images after 14 days of treatment with (**a**) phosphate-buffered saline (PBS), (**b**) α-CD-ss-Ce6 NPs without laser, (**c**) free Ce6 with laser, and (**d**) α-CD-ss-Ce6 NPs with laser. Reproduced from Reference [42] with permission from *Small*, 2016.

Redox-responsive capabilities of α-CD-ss-Ce6 micelles were explored, in vitro, through human oral epidermoid carcinoma (KB) cells pretreated with d,l-buthionine-sulfoximine (BSO), an inhibitor of intracellular synthesis of GSH, or with glutathione-reduced ethyl ester (GSH-OEt) to improve the intracellular GSH concentration. Upon 660 nm light irradiation (0.050 W, 2 min), KB cells pretreated with BSO exhibited lower viability inhibition compared to those with GSH-OEt. In vivo fluorescence imaging studies, conducted on KB tumor-bearing nude mice, revealed that the pseudopolyrotaxane nanocarrier has dramatically enhanced Ce6 accumulation and retention time in tumors compared to free chlorin e6, over 24 h. PDT experiments were performed in intravenously injected mice with phosphate-buffered saline (PBS) (control), α-CD-ss-Ce6 nanocarriers, or free chlorin e6 at a dosage of Ce6 2 mg/kg of total mouse body weight, and exposed to a 660 nm laser (0.300 W, 5 min) 4 h post-injection. Results demonstrated that free Ce6-treated mice did not show significant PDT efficacy after irradiation, while the α-CD-ss-Ce6 nanocarriers group exhibited remarkable tumor regression, Figure 8. After 14 days of treatment, a complete remission of tumors was observed in the group treated with α-CD-ss-Ce6, demonstrating excellent therapeutic effects after light irradiation.

## 3. Micellar Nanostructures for Nanotheranostics Based on Chemotherapy and Imaging

Manganese-based contrast agents were one of the first examples of paramagnetic contrast materials studied in magnetic resonance imaging (MRI). Manganese porphyrins are contrast agents that produce positive enhancement on T1-weighted MRI with several advantages over conventional agents (for example, Gd-DTPA), such as the high stability of the Mn(III)-porphyrin complex [43]. 

**Figure 9:**
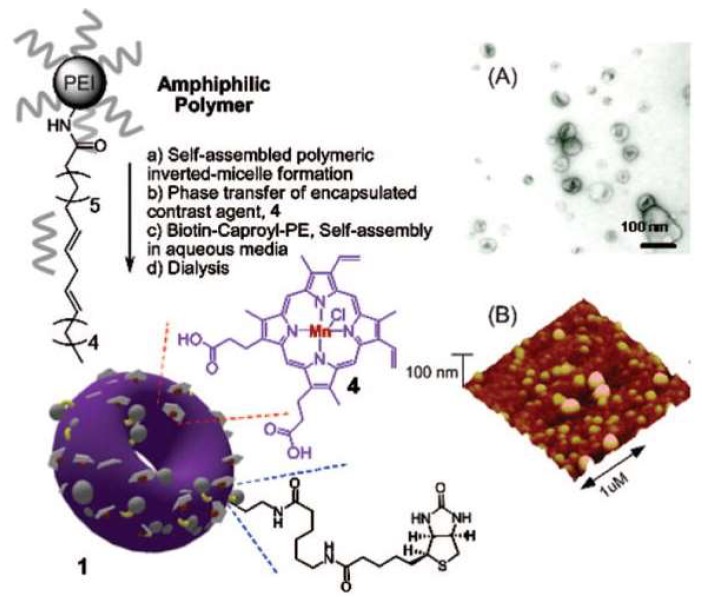
Schematic illustration of the synthesis, (**A**) transmission electron microscopy (TEM) and (**B**) atomic force microscopy (AFM) images of a Mn(III)-labeled nanobialy. 1. nanobialys and 4. Sensitizers numbered in the original figure. Reproduced from [44] with permission from *Journal of the American Chemical Society*, 2008.

Pan and co-workers devised a synthetic strategy consisting in the hydrophobic modification of PEIs with linoleic acid, by activation of the carboxylic acid moieties. Molecular self-assembly of those amphiphilic branched polyethylenimines produce inverted micelles characterized by a toroidal shape, called nanobialys, with tunable particle size and low polydispersity. Further synergistic self-assembly in the presence of biotin–caproyl–polyethylene lead to the bilayer structure depicted in Figure 9 [44]. Most importantly, the encapsulation of the water-soluble contrast agent Mn(III)-protoporphyrin IX chloride within these inverted micelles at the surface of the nanoparticles gives accessibility to water and removes detrimental interactions with surface-homing ligands or surrounding plasma proteins. The MRI of fibrin, a critical component of intravascular thromboses, was evaluated as follows: nanobialys with both biotin and metal, with biotin and no metal, or with metal and no biotin, were targeted to fibrin clot samples in vitro. The MRI results exhibited a pronounced contrast enhancement of the fibrin-targeted Mn(III) nanobialys and no contrast improvement from the non-targeted and metal-free nanobialys. The potential of this nanotheranostic agent for local delivery applications of chemotherapeutic agents was also assessed, in vitro, using doxorubicin and camptothecin (CPT); these matrices show high loading efficiencies (above 98%) and moderate release rates (ranging from about 12% to 20% over 3 days).

## 4. Micellar Nanostructures for Nanotheranostics Based on PDT Combined with Other Therapies and Imaging 

Combination therapy, the use of approaches that work by different mechanisms of action and affect different targets looking for an enhancement in overall therapeutic efficiency, is a common practice in many medical disciplines. PDT was used in combination with antioxidants, chemotherapy, immunotherapy, or photothermal therapy to enhance antitumor response [45,46]. Among the cancer therapies, photothermal therapy (PTT) can be highlighted. PTT is based on the use of compounds which transform the light absorbed in the NIR region into thermal energy, promoting local antitumor treatment. The requirements for the photothermal agent are coincident with some of the requirements for PDT sensitizers (biocompatibility and high absorption on the NIR region), therefore, the combination of both phototherapies was used to improve cancer treatment [47]. The combination of therapies could take advantages from the combination with imaging and the use of nanomaterials for delivery in the same way as single therapies. Herein are examples of nanotheranostics using combined therapies, imaging, and micelles.

**Figure 10:**
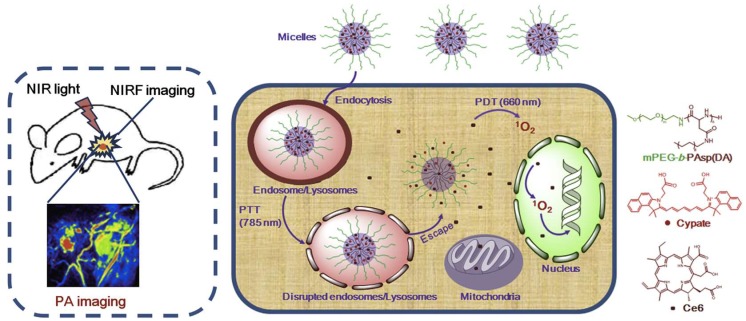
Schematic illustration of Ce6-loaded MPEG-*b*-PAspDA micelles incorporating cypate for dual-mode tumor photoacoustic (PA)/near-infrared fluorescence (NIRF) imaging and synergistic cancer photothermal therapy (PTT)/PDT through enhanced cytoplasmic delivery of the photosensitizer. Reproduced from Reference [48] with permission from *Biomaterials*, 2014.

Preparation of a copolymer composed of MPEG and decylamine-grafted poly(l-aspartic acid) (MPEG-*b*-PAspDA) allowed the preparation of micelles for the encapsulation of chlorin e6 (Ce6) and cypate (Cy). The incorporation of the sensitizer and the dye—highly fluorescent in the infrared region—into the micelles opened the way to developing nanotheranostic platforms for precise tumor localization via dual photoacoustic (PA)/near-infrared fluorescence (NIRF) imaging and, simultaneously, more efficient cancer treatment via sequential and synergistic photothermal therapy (PTT)/photodynamic therapy (Figure 10) [48]. The preparation of the copolymer MPEG-*b*-PAspDA was achieved through the ring-opening polymerization of β-benzyl _L_-aspartate *N*-carboxyanhydride with 12 kDa MPEG and subsequent aminolysis of decylamine. Mixing Ce6, Cy, and the copolymer in dimethylsulfoxide, with further dispersion into water and dialysis, yielded micelles with high loading capacity (20% (*w*/*w*)) for both Ce6 and Cy. TEM imaging indicated that the micellar structures had a spherical shape and an average size of 55 nm, smaller than dimensions obtained by DLS (63.6 nm). Furthermore, stability of these micelles at pH = 7.4 protect Ce6 from chemical degradation at physiological pH, due to its effective encapsulation within the micellar cores. The performance of Cy/Ce6-micelles towards cellular internalization, photostability, and PTT and PDT activity were also evaluated, in vitro, using 4T1 murine breast cancer cell lines.

Cy/Ce6-micelles were intravenously injected into mice bearing 4T1 murine tumors and the NIRF intensities of Cy were evaluated ex vivo at 24 h post-injection. Cypate released from the micellar systems was mainly accumulated in the tumor, liver, and kidneys. The higher accumulation of Cypate in the tumor area observed in mice injected with Cy/Ce6-micelles, when compared with observations using free Cy/Ce6, was attributed to the enhanced permeability and retention effect of the micelles. However, free Cy/Ce6 exhibited high accumulation into the liver, which can be explained by the absence of the targeting effect. To further validate the capacity of Cy/Ce6-micelles for cancer imaging purposes, in vivo NIRF imaging and PA imaging on 4T1 tumor-bearing mice were assessed. Cy/Ce6-micelles revealed a significant tumor buildup of NIRF signals from cypate 24 h after injection, in agreement with its biodistribution. In particular, Cy/Ce6-micelles presented relatively prolonged retention of NIRF signals in the tumor during the imaging period of 4 days, compared to free Cy/Ce6, accompanied by low NIRF noise from some main normal tissues, such as lung, kidneys, and spleen, 24 h after injection. The enhanced accumulation of cypate in tumor tissue and its rapid scavenging from normal tissue accounts for the high signal-to-noise ratio at the tumor site, which is a key factor to achieving an ultrasensitive tumor tissue detection. Regarding PA imaging, the tumor presented weak PA signals before injection, and only large tumor vessels were detected. However, PA imaging displayed a clear 3D tumor microstructure, with high contrast and spatial resolution, 6 h after injection of the Cy/Ce6-micelles. The PA signals at the tumor are further enhanced at 24 and 48 h post-injection. Furthermore, the improved signals 24 h after injection shown a uniform distribution of cypate within the microvessels of the tumor, which implies that Cy/Ce6-micelles are capable of triggering effective photothermal and photodynamic damage on both tumor cells and tumor vasculature, upon irradiation. The control groups, phosphate-buffered saline (PBS) and free Cy/Ce6-injected mice, presented negligible increase of PA signals even 48 h after injection.

Attempting to elucidate the in vivo efficacy of Cy/Ce6-micelles by synergistically integrating PTT and PDT, 4T1 tumor-bearing mice were injected with a 7.5 mg/kg dose, followed by treatment with various light irradiation modalities 24 h after injection; tumor volumes were then monitored for up to 16 days. The PBS control group displayed a 9-fold increase of average tumor volumes compared to their original volumes, regardless of irradiation. Cy/Ce6-micelles under PTT treatment, with irradiation at 785 nm, exhibited a substantial photothermal damage triggering tumor necrosis 2 days after injection, which resulted in a 5.4-fold increase of tumor volumes after 16 days. A 7-fold increase of tumor volumes under PDT treatment with irradiation at 660 nm was also driven by Cy/Ce6-micelles. Noticeably, the photodynamic activity of Cy/Ce6-micelles exhibited a smaller tumor inhibition effect as compared to their photothermal damage, which was explained due to insufficient cytoplasmic distribution of the sensitizes and narrower tissue penetration depth of 660 nm light. Most interestingly, Cy/Ce6-micelles caused tumor necrosis at 2 days post-injection and did not produce significant tumor growth during 16 days under sequential PTT/PDT treatments, thus achieving a superior tumor inhibition effect. By contrast, Cy/Ce6-micelles exposed to individual PDT/PTT actions displayed less tumor inhibition efficacy than those under the sequential PTT/PDT treatments. Hence, successive PTT/PDT treatments resulted in a synergistic efficacy rather than a combinatorial effect; probably, the PTT-triggered disruption of lysosomal membranes contributes to the synergistic effectiveness through the enhanced cytoplasmic delivery of Ce6, which provides an effective pathway to transport more PS to cytosol and targeted organelles, improving the susceptibility of cancer cells to the PS and, lastly, facilitating the synergistic consequence of PTT and PDT. Since NIR light can successfully penetrate into inaccessible tumors, these theranostic micelles present a high potential to treat small sized-tumors during a prolonged imaging period, after accurate detection and localization, with reduced adverse side effects in healthy tissues. Cy/Ce6-micelles caused stark cancer necrosis with less cancer cells and wide hemorrhagic inflammation under PTT/PDT, as compared to those under PDT/PTT, whereas those under PTT or PDT treatment simply exhibited hemorrhagic inflammation and sporadic necrotic region surrounded by malignant cells with nuclear atypia.

**Figure 11:**
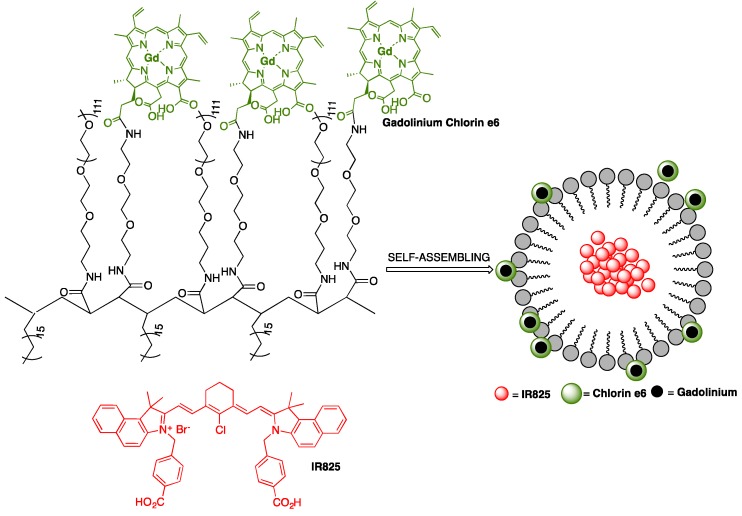
Schematic illustration of IR825@C18PMH-PEG-Ce6-Gd nanomicelles; Ce6 is attached on the backbone of C18PMH-PEG polymer via a short PEG linker; Gd^3+^ forms a chelate complex with Ce6; IR825 is encapsulated inside the formed micellar system.

A multifunctional polymeric nanomicelle system containing Ce6 and near-infrared dye IR825 was conceived by Gong and co-workers for multimodal imaging-guided combined photothermal and photodynamic therapy applications [49]. Poly(maleic anhydride-*alt*-1-octadecene) (C18PMH) was primarily reacted with a long PEG-amine (5 kDa) and a short diamine-PEG (324 Da), yielding C18PMH-PEG_5k_/PEG_324_-NH_2_, followed by conjugation with Ce6. The obtained Ce6-grafted amphiphilic polymer (C18PMH-PEG-Ce6) was then used to encapsulate IR825, producing IR825@C18PMH-PEG-Ce6 nanomicelles that comprise both photosensitizer and photothermal agents, Figure 11. The loading of Ce6 and IR825 were 3.8% and 10%, respectively. The encapsulation of IR825 leads to a significant increase in the size of C18PMH-PEG-Ce6 micelles. From DLS measurements, it was possible to conclude that micelles size increase from about 26 nm (without dye) to around 107 nm. This is in agreement with SEM images, showing micelles with uniform morphology and size diameters ranging between 100 and 200 nm. The carboxyl groups on Ce6 promote negatively charged micelles, which were characterized by a zeta potential of −9 mV. The micellar structures are highly stable in various physiological solutions, without substantial release of Ce6 or IR825, even after 7 days of incubation with PBS. In addition, Ce6 was used to chelate the commonly used MRI contrast agent Gd(III) for the measurement of the spin-lattice relaxation time *T*_1_; the chelation process of Gd(III) by Ce6 linked to the polymer support occurred at a molar ratio of 1:1, as demonstrated by inductively coupled plasma atomic emission spectroscopy (ICP-AES) studies, after removal of unbound Gd(III) through dialysis. To explore the opportunity of using IR825@C18PMH-PEG-Ce6-Gd as a *T*_1_-weighted MRI contrast agent, its *T*_1_ relaxation time as a function of Gd(III) concentration was evaluated. The T_1_ value for Ce6 chelated Gd(III) (Ce6-Gd) was estimated to be 1.95 mM^−1^s^−1^, which was lower than that of the clinically approved contrast agent Magnevist (4.29 mM^−1^s^−1^). Nonetheless, the T_1_ value of IR825@C18PMH-PEG-Ce6-Gd was 29.91 mM^−1^s^−1^, i.e., approximately 7 times larger than that of Magnevist. The singlet oxygen generation capability of Ce6 was slightly quenched after chelation with Gd(III), however, no considerable variance was observed between IR825@C18PMH-PEG-Ce6 and IR825@C18PMH-PEG-Ce6-Gd, which allowed the use of these nanomicelles in combined PDT/PTT or PTT/PDT experiments, as well as in multimodal imaging, both in vitro and in vivo. 

Mice bearing 4T1 tumor cell lines with initial volumes of 100–150 mm^3^ were selected and arbitrarily divided into 5 groups, one being injected with saline as a control and the other four with IR825@C18PMH-PEG-Ce6-Gd (200 μL, [Ce6] = 0.5 mg/mL, [IR825] = 1.3 mg/mL); 12 h after injection, PTT treatment, PDT treatment and treatments using a combination of PTT/PDT and PDT/PTT were conducted and analyzed. For the PTT treatment, irradiation was carried out with 808 nm light at a 0.3 W/cm^2^ power density and the tumor temperature kept at 49 °C for 6 min; this showed tumor growth was mainly repressed in the first 8 days, after which rapid regrowth occurred. The PDT treatment consisted of irradiation with 660 nm light at a 2 mW/cm^2^ power density for 1 h, inhibiting tumor development only partially. The therapeutic efficiencies for both the PDT/PTT and PTT/PDT treatments displayed no relevant differences, suggesting that sequence was not a key issue in the combination therapy. In addition, no substantial toxicity of the nanotheranostic micelles was found.

**Figure 12:**
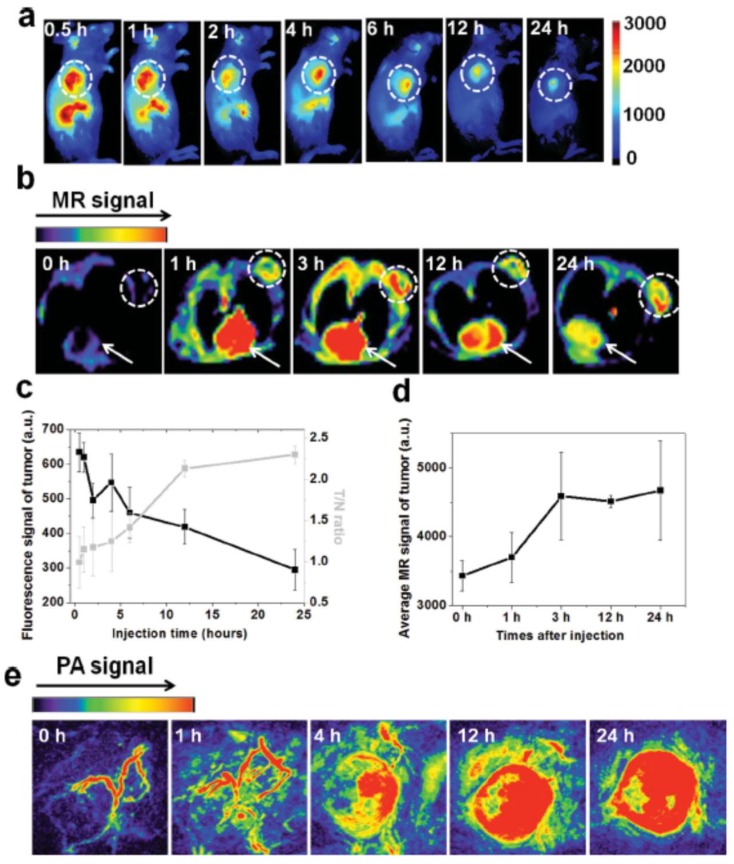
(**a**) In vivo fluorescence imaging and (**b**) MRI of mice bearing 4T1 tumor cells taken at different time points post-injection with IR825@C18PMH-PEG-Ce6-Gd (200 μL, [Ce6] = 0.5 mg/mL, [IR825] = 1.3 mg/mL); dashed circles in (**a**) and (**b**) highlight the tumor, while arrows in (**b**) point to the heart; (**c**) quantification of tumor signals and tumor-to-normal tissue signal (T/N) ratios from fluorescence images shown in (**a**) at different post-injection times; (**d**) quantification of average T1-MRI signals in the tumor by manual drawn region of interest at different post-injection times; (**e**) in vivo PA imaging of mice bearing 4T1 tumor cells taken at different time points post-injection of IR825@C18PMH-PEG-Ce6-Gd. Reproduced from Reference [49] with permission from *Advanced Functional Materials*, 2014.

IR825@C18PMH-PEG-Ce6-Gd micelles can be used as a multifunctional imaging agent that offers contrast in three different modalities: fluorescence imaging, MRI, and PA imaging. Ce6 fluorescence was recorded, in vivo, on 4T1 tumor-bearing mice at different time points after injection with IR825@C18PMH-PEG-Ce6-Gd to measure the tumor-to-normal tissue signal (T/N) ratio and determine the ideal time window for the ensuing therapy. The fluorescence signals reached a peak value 4 h after injection, Figure 12a,c, indicating the gradual accumulation of the micellar nanostructures in the tumor; 12 h after injection, the T/N ratio reached its maximum value and was steadily retained at that level, Figure 12c. IR825@C18PMHPEG-Ce6-Gd micelles were also tested for *T*_1_-weighted MRI, Figure 12b, which shows the cross-section *T*_1_ MRI images of mice acquired at different time intervals post-injection. The brightened *T*_1_ MRI signals in the tumor appeared early and gradually reached a plateau 3 h after injection. Contrarily to the Ce6-based fluorescence imaging, the MRI signals in the tumor remained quite stable after 3 h, Figure 12d; this divergence might be explained by the fluorescence-quenching effect of Ce6 over time in the mouse body. Apart from tumor imaging, this contrast agent appeared to be suitable for blood pool imaging, given that the heart muscle could be noticeably visualized under MRI conditions after injection of the IR825@C18PMHPEG-Ce6-Gd micelles, and the signal persisted even after 24 h, Figure 12b. PA images were also obtained at various time points post-injection of IR825@C18PMH-PEG-Ce6-Gd, Figure 12e. Before injection, only large blood vessels could be envisioned under PA imaging conditions due to the endogenous hemoglobin in the blood. However, after injection, the IR825-containing micellar structures became homogenously distributed inside the tumor due to passive accumulation, allowing the PA signal to grow with time.

**Figure 13:**
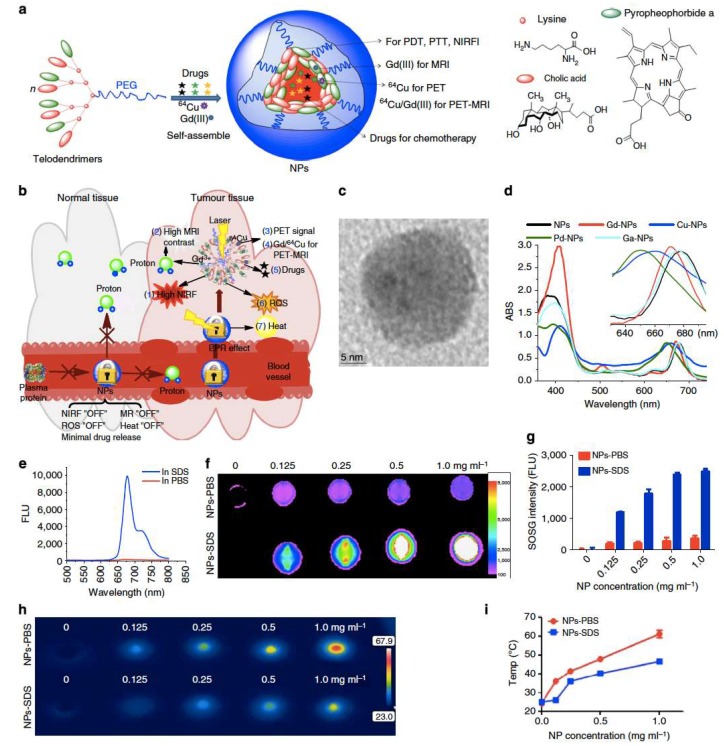
(**a**) Schematic illustration of a multifunctional NPor self-assembled by a representative porphyrin-telodendrimer system, PEG_5k_-Por_4_-CA_4_, comprised of four Por molecules and four CA moieties, attached to the terminal end of a linear PEG chain and (**b**) its use as a “all-in-one” nanomedicine platform against cancer; (**c**) TEM image of NPors; (**d**) absorbance spectra of NPors before and after chelating different metallic ions; (**e**) fluorescence emission spectra of NPs in the presence of PBS and SDS using a 405 nm excitation; (**f**) NIRF imaging of a NPor solution in the presence and absence of SDS with an excitation bandpass filter at 625/20 nm and an emission filter at 700/35 nm; (**g**) single oxygen generation of NPs in PBS and SDS after light irradiation (690 nm at 0.25 W/cm^2^ for 60 s); concentration-dependent photothermal transduction of NPors—(**h**) thermal images and (**i**) quantitative temperature change curve; the temperature of the NPor solution in the presence and absence of SDS was monitored by a thermal camera after irradiation with NIR light (690 nm at 1.25 W/cm^2^ for 20 s). Reproduced from Reference [50] with permission from *Nature Communications*, 2014.

A hybrid polymer made by self-assembly of a novel class of amphiphilic polymers (telodendrimers), comprising linear PEG, dendritic oligomers of pyropheophorbide-a (Por), and cholic acid, was used as a single organic building block to prepare a porphyrin-based platform (NPor). These porphyrin base nanostructures are capable of several imaging and therapeutic functionalities, such as NIRF imaging, PTT, or PDT. Moreover, taking advantage of the inherent capability of the porphyrins at the polymer structure to form complexes with several metal ions, namely ^64^Cu or Gd(III), the Npor could be use as agents for positron emission tomography (PET) imaging and MRI, Figure 13a,b [50]. DLS and TEM experiments characterized the particles as spherical in shape with size around 20 nm, Figure 13c. The NPors and metal ion-loaded NPors generated by the self-assembly of metal-complexed telodendrimers present very interesting photophysical and photochemical properties. In PBS, Npor and their metal complexes displayed two absorption bands, one around 405 nm and other at the NIR region between 650 and 690 nm, Figure 13d; both fluorescence and capability of ROS generation are highly self-quenched, and the absorbed energy after irradiation is released in the form of heat increasing the temperature of the surrounding media with the increasing of the NPor concentration, Figure 13h,i. The addition of sodium dodecyl sulfate (SDS) promotes micellar dissociation, increasing significantly the near-infrared fluorescence intensity at 680 nm, Figure 13e,f, and, consequently, will led to re-establishment of the ROS generation capability, Figure 13g, and promoting a less noteworthy rise in the solution temperature. This architecture-dependent “on/off” fluorescence and ROS generation and photothermal transduction is the base for the expected enhancement of fluorescence signal at the tumor sites and/or inside the tumor cells, and increased therapeutic effect. 

In an attempt to further increase the structural stability of NPors in blood circulation, the same authors employed a reversible disulfide crosslinking approach. Four cysteine (Cys) units were introduced to the backbone of the telodendrimer, PEG_5k_-Cys_4_-Por_4_-CA_4_ being prepared; the resulting NPors were then crosslinked via disulfide bond through oxidation of the thiol groups on the cysteine residues. TEM studies indicated that these crosslinked nanostructures (CNPors) were spherical vesicles of 32 ± 8 nm in diameter. The success of this strategy was confirmed by the longer circulation time observed in vivo for CNPors when compared with NPors. Moreover, using Gd(III)-chelated CNPors and NPors, it was possible to conclude that Gd-CNPors accumulated 2.8 and 12.3 times more in tumor (SKOV3 ovarian cancer xenograft) than Gd-NPors and Gd-DTPA, respectively. The fluorescence signal at the tumor was 15.2 times higher than in muscle and 3.1 times higher than the signal obtained with NPors at the tumor site. The architecture-dependent “on/off” fluorescence capability was retained. In vivo experiments in nude mice bearing a SKOV3 ovarian cancer xenograft showed that the fluorescence signal in the whole body decreased using CNPors in contrast to NPors. However, the cleavage of disulfide bonds by GSH at the tumor site resulted in the amplification of the fluorescence signal. 

**Figure 14:**
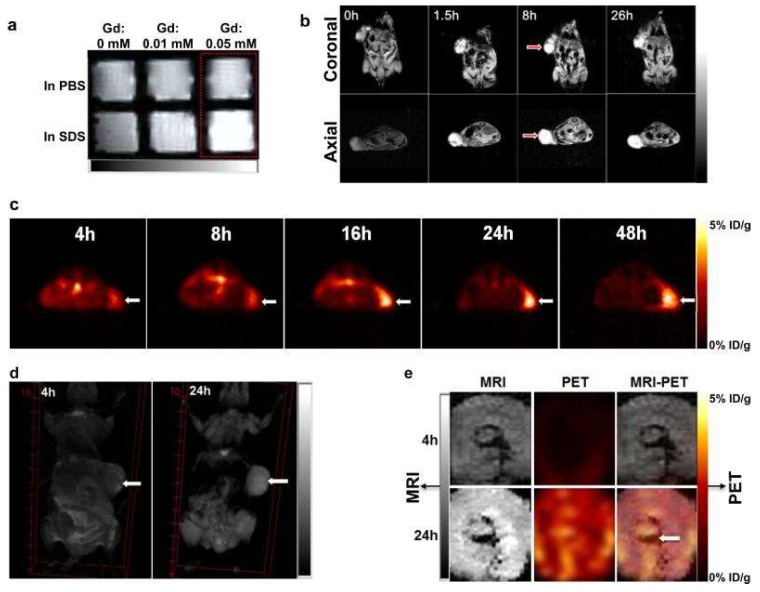
NPor-mediated MRI and PET imaging in animal models. (**a**) MRI signal of Gd-NPors in the presence and absence of SDS obtained in vitro by *T*_1_-weighted MRI; (**b**) representative coronal and axial MRI images of transgenic mice with mammary cancer (FVB/n Tg(MMTV-PyVmT)) at pre-injection and post-injection of 0.15 mL Gd-NPors (Gd dose = 0.015 mmol/kg); red arrows point to the tumor site; (**c**) PET image of nude mice bearing SKOV3 ovarian cancer xenografts at 4, 8, 16, 24, and 48 h post-injection of ^64^Cu-NPors (150–200 µL, ^64^Cu dose = 0.6–0.8 mCi); white arrows point to the tumor site; (**d**) 3D coronal MRI images of nude mice bearing A549 lung cancer xenografts at 4 or 24 h post-injection with 0.15 mL of ^64^Cu and Gd dual-labeled NPors (150–200 µL, ^64^Cu dose = 0.6–0.8 mCi, Gd dose = 0.015 mmol/kg); white arrows point to the tumor site; (**e**) PET-MRI images of tumor slices of nude mice bearing A549 lung cancer xenografts at 4 or 24 h post-injection of dual-labeled NPors; white arrows point to the necrotic area in the center of the tumor. Reproduced from Reference [50] with permission from *Nature Communications*, 2014.

Gd-NPors also exhibit architecture-dependent magnetic resonance properties that allow their use as contrast agents for sensitive and tumor-specific MRI. There is low MRI signal enhancement when Gd-NPors retain their micellar structure, Figure 14a, probably because of Gd/Por stacking sheltering Gd(III) from interacting with protons in water. However, upon micellar dissociation, gadolinium ions have access to neighboring protons, resulting in an improved MRI signal. This distinctive feature of Gd-NPors offers sensitive MRI detection of tumor tissues. The application of Gd-NPors for MRI in transgenic mice with mammary cancers shows considerable improvement of the contrast of the signal of tumors 1.5 h after injection, being sustained for more than 26 h, Figure 14b, while in normal tissues, only a very low signal improvement was observed. To extend the capabilities and to be able to use NPors for PET imaging, ^64^Cu(II) radiotracer was incorporated into the NPors with radiochemical yield over 96.5%. ^64^Cu-NPors started to accumulate within SKOV3 ovarian tumor cells in nude mice at 4 h post-injection, with a maximum level being reached at 16 h. After 24 h, the radiolabel was found mainly at the implanted tumors, with quite low background signals in the rest of the body, Figure 14c. The dual-labeled NPors obtained by integration of both ^64^Cu(II) and Gd(III) greatly enhance the MRI contrast and PET signal within A549 lung cancer xenografts at 24 h post-injection, Figure 14d,e. Remarkably, the heterogeneity of the tumors and the homogeneous distribution of NPors within them were noninvasively exposed using PET-MRI, Figure 14e, proving the capabilities of NPors as dual-modality nanoprobes for PET-MRI.

In addition to their capabilities as imaging agents, NPors and their crosslinked derivatives CNPors could be applied for the improvement of therapies. CNPors could effectively serve as carriers for poorly water-soluble chemotherapeutic drugs. For example, DOX could be encapsulated inside CNPors with a loading capacity of 2.90 mg/mL, producing DOX-loaded CNPors (CNPOR-DOX) that are highly stable in human plasma for 24 h at 37 °C. Fluorescence resonance energy transfer (FRET) screening and dialysis experiments pointed out that DOX release is sluggish in human plasma, but could be assisted through illumination, probably as a result of local heat formation, or addition of GSH at intracellular levels (10 mM). 

PTT and PDT treatments were performed in transgenic mice with mammary cancer 24 h after CNPors injection. The temperature at the tumor site increased to 57 °C after light irradiation (1.25 W/cm^2^ for 2 min), in contrast with 41 °C in the PBS control group, which is sufficient to cause permanent damage to tumor cells. ROS generation by CNPors after irradiation was also considerably higher than the tissue background in the PBS control group. Histopathology studies revealed big areas of severe tissue damage, as a result of cellular destruction and apoptosis, which can be ascribed to antitumor effects from PTT and PDT treatment. Drug-loaded CNPors are projected to release drugs and generate singlet oxygen and heat in a simultaneous manner upon irradiation with NIR light for concomitant PTT/PDT and chemotherapy applications. In fact, an in vivo experiment using mammary cancer-bearing mice, aimed to assess the antitumor effectiveness of CNPors in transgenic and xenograft mouse models, was performed. CNPor-DOX-mediated PTT/PDT was found to significantly hinder tumor growth when a light dose of 1.25 W/cm^2^ for 2 min was applied once a week, compared with the PBS control group and the normal micelles without porphyrin group. The treated tumors were entirely eradicated on day 12 with ulceration, and no palpable new tumors were detected even on day 32. CNPor-DOX-promoted combination therapy of PTT/PDT with doxorubicin showed a similar efficacy, lower doses of CNPors, and/or lower light dosages should be employed in order to effectively establish a comparison of the efficacy of the several CNPor-mediated combination therapies. In nude mice bearing SKOV3 ovarian cancer xenografts, the CNPor-promoted phototherapy, DOX alone, and NM-DOX-treated groups all caused a slower tumor growth when compared to the PBS control group. Utilizing the same dose of DOX, PS, and light (0.25 W/cm^2^ for 2 min), the combination treatment with CNPor-DOX disclosed the best antitumor activity, with a total inhibition of tumor growth being observed throughout the study. Moreover, no substantial variations in body weight, blood count, and serum chemistry were detected after three doses of treatment. MRI was also engaged to verify the tumor growth after administration of Gd-NPors with and without light irradiation. MRI images at 48 h post-injection (24 h after irradiation) displayed both reduction of the tumor and rise in necrotic volume at the tumor site. MRI demonstrated total tumor elimination at 7 days post-injection in contrast to no variance in tumor size being perceived in control mice that were not subjected to light irradiation.

Photosensitizer Ce6 and chemotherapeutic agent doxorubicin were used by Hou et al. to prepare pH-sensitive theranostic NPs for tumor NIR fluorescence imaging and chemo–photodynamic combination therapy [51]. Nanoparticles (TCAD) were obtained via self-assembly in aqueous solution of the acid-sensitive *cis*-aconitic anhydride-modified doxorubicin (CAD) conjugated to D-α-tocopheryl poly(ethylene glycol) 1000 succinate (TPGS) and subsequent loading of Ce6-originated micelles incorporating the chemotherapy agent and the sensitizer (TCAD@Ce6 NPs). The best ratio for Ce6/conjugate TPGS-CAD was found to be 1:20 (*w*/*w*), and, under these optimized conditions, the encapsulation efficiency, loading efficacy, and hydrodynamic diameter were 85.25%, 14.89%, and 160 nm, respectively. Analysis by TEM and field emission scanning electron microscopy revealed that they are almost spherical with good monodispersity. A considerable release of chlorin e6 and DOX from the micellar system to the aqueous medium occurred under acidic conditions (pH 6.5 and 5.5), mimicking the tumor and intracellular microenvironment. This was attributed to hydrolysis of the acid-sensitive amide linker present in TCAD@Ce6 NPs. The cellular uptake behavior of TCAD and TCAD@Ce6 nanoparticles was evaluated by confocal laser scanning microscopy and flow cytometry toward non-small cell lung carcinoma (A549 cells). Fluorescence data have shown that the uptake by cells was higher when incubated (4 and 12 h) with NPs than with the free form of DOX and Ce6. This could explain the better antiproliferation efficacy of TCAD NPs on A549 cells, in the absence of light, than that of the equivalent free DOX, after 24 or 48 h co-incubation. In vitro efficiency of chemo–photodynamic combination therapy of TCAD@Ce6 NPs to A549 cells was quantified by MTT assay. Thus, upon irradiation (633 nm HeNe laser) at a power of 50 mW/cm^2^ for 3 min, after 24 h incubation, the survival ratio of A549 cells decreased to 15.73% when exposed to TCAD@Ce6 NPs (equivalent Ce6 4.0 μg/mL and equivalent DOX 5.86 μg/mL). Under similar conditions, the survival ratio of A549 cells was 71.2% and 66.44% when treated with free Ce6 (4.0 μg/mL) and TCAD NPs (equivalent DOX 6.0 μg/mL), respectively. In vivo studies in A549 tumor-bearing nude mice confirmed TCAD@Ce6 NPs as efficient theranostic agents for cancer. The mean fluorescence intensity accumulation in the tumors of Ce6 in TCAD@Ce6NPs was about 18-fold higher than free Ce6 (12 h post-injection) and exhibited relatively long tumor retention time. Furthermore, upon NIR laser irradiation (633 nm, 50 mW/cm^2^, 30 min), 12 h after injection, significant tumor growth suppression was achieved when mice were treated with TCAD@Ce6NPs. The chemo–photodynamic combination therapy with this prodrug micellar system was shown to be much more efficient than chemo or photodynamic treatments with DOX and Ce6 alone, respectively.

**Figure 15:**
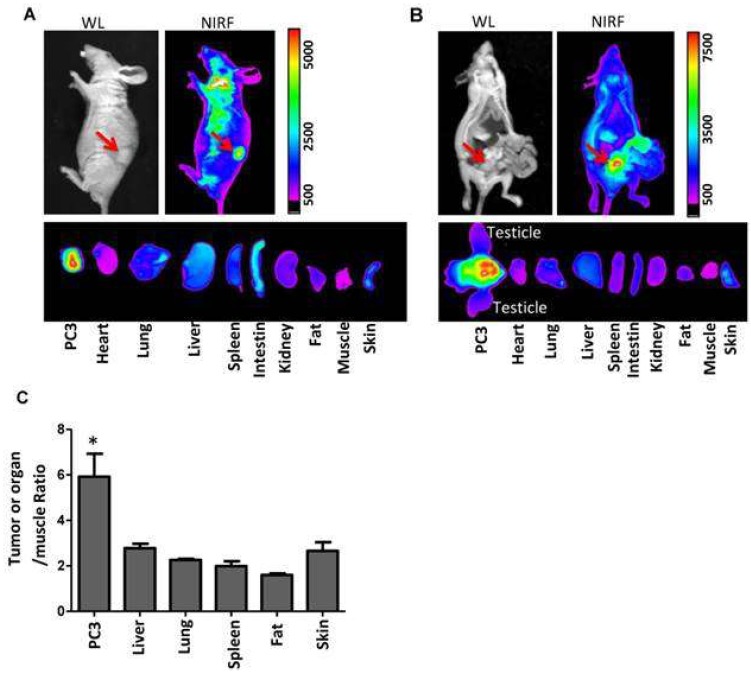
Imaging-guided drug delivery of NP-AAG in both mouse models bearing (**a**) subcutaneous and (**b**) orthotopic PC3 xenograft. In vivo and ex vivo white light (WL) and NIRF imaging of nude mice bearing subcutaneous PC3 xenograft (a) or orthotopic PC3 xenograft (b) 72 h post-injection of NP-AAG. Arrows: tumor site. (**c**) Analysis of NIRF imaging on each tumor and organs after normalization to the fluorescence of muscle. Reproduced from Reference [52] (*Theranostics* 06: 1324 image No. 006, open access policy).

A novel multifunctional nanomicellar-platform for targeted combination therapy against prostate cancer, photothermal, and photodynamic therapies (PTT/PDT), was reported by Lin and Guo et al. [52]. They have developed porphyrin-based telodendrimers comprising polymers of linear PEG and oligomers of pheophorbide-a. After self-assembly under aqueous conditions, 17-allylamino-17-demethoxygeldanamycin (17AAG) was loaded into micelles. This type of molecule could act as a heat shock protein 90 (Hsp90) inhibitor, decreasing the levels of pro-survival and angiogenic signaling molecules induced by phototherapy. The 17AAG loading efficiency into micelles determined by HPLC was approximately 100%, and the analysis of the particle size (NP-AAG) was shown by DLS to be about 22 nm. Their morphology, as observed by TEM, was spherical. It has been demonstrated that this nanoporphyrin-based drug delivery system can be triggered by NIR fluorescence for optical imaging in nude mice bearing either a subcutaneous or orthotopic PC3 prostate cancer xenograft. It was also observed that PC3 cancer cells, Figure 15, could efficiently internalize NP-AAG. After 2 h of incubation, NP-AAG was distributed mainly in the cytoplasm. The synergetic effect of 17AAG and nanoporphyrin-mediated PDT was evaluated in two prostate cancer cell lines, androgen-dependent (LNCAP) and -independent (PC3), and in normal prostate cells (RWPE1). Compared to normal prostate cell RWPE1, both PC3 and LNCAP cells were more sensitive to nanoporphyrin-mediated PDT/PTT, while there were no cytotoxicity effects observed in all cells treated with NP without light. Moreover, under irradiation (NIR light at 0.07 W/cm^2^ for 2 min), MTT assays with these cancer cell lines have clearly demonstrated that the incorporation of 17AAG into NPs improved the PDT/PTT efficiency. Further studies in PC3 cells showed that NP-AAG promote a decrease of the levels of phototherapy-induced pro-survival and angiogenic proteins (HIF1α, survivin, AKT, MMP2, Src), thus enhancing the anticancer efficacy. The authors extended the study to animal models. Experiments in nude mice bearing subcutaneous PC3 have shown that combination therapy with NP-AAG was more efficacious than single treatment modality with identical dose of drug and light, resulting in prolonged survival times.

Ce6 was also chosen by Zhang et al. as a sensitizer in multifunctional lipid-micelles obtained from self-assembly, in aqueous solution, of a mixture of MRI agent Gd-DOTA conjugated with [poly(ethylene glycol) 2000]-2-distearoyl-*sn-glycero*-3-phosphoethanolamine (lipid–Gd-DOTA), incorporating semiconducting polymer dots (Pdots) of poly[2,6-(4,4-bis-(2-ethylhexyl)-4*H*-cyclopenta[2,1*-b*;3,4*-b’*]-dithiophene)-*alt*-4,7-(2,1,3-benzothiadiaxole)] (Pdots/Ce6@lipid–Gd-DOTA) for combined MRI/PA imaging and combined PDT/PTT treatment [53]. The semiconducting polymer dots and Ce6 molecules were co-loaded into the micelles at 16 and 24 wt %, respectively. DLS analysis showed that the average hydrodynamic size of the micelles was 111.5 nm and the polydispersity index 0.289. MRI contrast-enhancement capability of these Pdots/Ce6@lipid–Gd-DOTA micelles on a 9.4 T MRI system was determined in tumor tissues (HepG2 tumor-bearing nude mice). Uptake of Pdots/Ce6@lipid–Gd-DOTA reaches a maximum value after 3 h of administration, determining the time of incubation before PDT/PTT treatment. Very few cytotoxic effects were observed without laser irradiation on the NIH-3T3 cells incubated with Pdots/Ce6@lipid–Gd-DOTA micelles for 48 h. However, dose-dependent PDT/PTT activity was verified under 670 nm laser irradiation (0.5 W/cm^2^); after irradiation, the viability of HepG2 cells treated with Ce6 (2.4 mg/mL) or Pdots@lipid-micelles (Pdots, 1.6 mg/mL) sharply decreased to 25.6% and 44.7%, respectively. Pdots/Ce6@lipid–Gd-DOTA micelles with the same concentration of sensitizer and dots achieve a more pronounced effect, with HepG2 cells showing a viability of 15.3%. This synergistic PDT/PTT effect was evaluated, in vivo, using mice bearing HepG2 tumor. Under irradiation, no significant change of temperature occurred in tumors treated with Ce6 while, in mice treated with Pdots/Ce6@lipid–Gd-DOTA micelles, an increase of temperature to approximately 52 °C at the tumor site was detected. The Pdots/Ce6@lipid–Gd-DOTA-micelles-treated group exhibited much higher therapeutic efficiency when compared with the Ce6-treated or Pdots@lipid-micelles-treated group alone, promoting an almost completely restrained growth of the tumor over 19 days.

**Figure 16:**
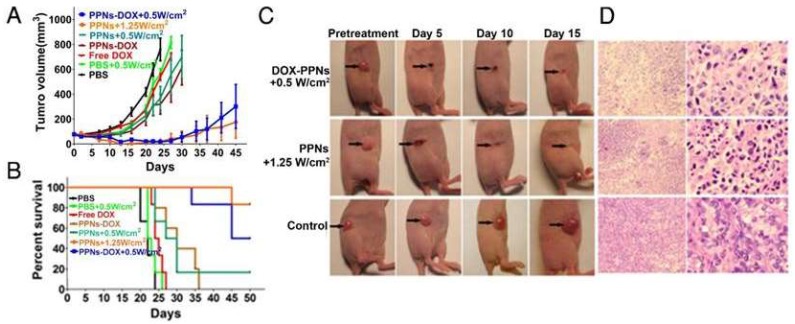
In vivo therapeutic response to PPNs-mediated chemotherapy combined with phototherapy. (**A**) In vivo antitumor efficacy after the intravenous treatment of various DOX formulations combined with PPNs-mediated phototherapy (*n* = 6). The SKOV3 tumor-bearing mice were intravenously injected with PBS (control), free DOX (2.5 mg/kg), PPNs (2 mg/kg calculated on porphyrin), and PPNs–DOX (PPNs 2 mg/kg, DOX 2.5 mg/kg) on day 0, 5, 10, and 15, followed by various light irradiations for 2 min on tumors, 24 h post-injection. (**B**) Kaplan–Meier survival curves of SKOV3 tumor-bearing mice treated with the above indicated conditions, where tumor volume reached 500 mm^3^ and was considered as the end point of survival data. (**C**) Photographs showing therapeutic response to PPNs-mediated phototherapy with irradiation at 0.5 W/cm^2^ for 2 min and 1.25 W/cm^2^ for 2 min, respectively. (**D**) H&E staining of tumor sections collected from control mice and PPNs-injected (2 mg/kg) and variously irradiated mice (0.5 W for 2 min and 1.25 W for 2 min, respectively), 24 h post-injection. Reproduced from Reference [54] (*Theranostics* 07:3901 image No. 005, open access policy).

More recently, Li et al. have described poly(vinyl alcohol)-porphyrin-based nanotheranostics (PPNs) that integrate the optical features for NIR and PET imaging, and the properties for therapeutics using PDT and PTT [54]. Poly(vinyl alcohol)-based micelles, incorporating the sensitizer pyropheophorbide-a (Por), chemotherapeutic drug doxorubicin, and PET imaging agent ^64^Cu, were prepared by a “one-pot” procedure. Poly(vinyl alcohol) (PVA) conjugated to pyropheophorbide-a self-assembled to form PPNs in aqueous environment with the hydrophobic porphyrin core surrounded by hydrophilic PVA chains and π–π stacking between DOX and porphyrin. The morphology in PBS of PPNs observed under TEM exhibited spherical shapes and relatively uniform size distribution. The particle size of PPNs determined by DLS was 78 nm. Intracellular uptake behavior and localization of DOX and PPNs–DOX were examined in SKOV3 ovarian cancer cells. After 6 h of incubation, total DOX fluorescence emission was detected inside the nuclei and the porphyrin fluorescence signal was strong in the cytoplasm, therefore showing the intracellular distribution of PPNs–DOX. The combination of irradiation and chemotherapy led to a significantly more effective in vitro toxicity against SKOV3 ovarian cancer cells than free DOX, PPNs–DOX without light, or PPNs-mediated PDT, taking advantage of both chemo- and photodynamic therapies for cell killing. NIRF allowed the in vivo determination of the maximum accumulation in tumor tissue (mice bearing SKOV3 ovarian cancer xenografts) 18 h after injection with PPNs–DOX. Additionally, PET imaging analysis showed that ^64^Cu-labeled PPNs started to accumulate at tumor sites 16 h after injection. The enhanced in vivo antitumor efficacy through synergistic PPNs–DOX-mediated combination of phototherapy (low light dose, 0.5 W/cm^2^ for 2 min) and chemotherapy was observed as a remarkable delay in tumor growth. Mice that received a high light dose, 1.25 W/cm^2^ for 2 min, developed eschars on tumors, starting from day 2 post-treatment, and the tissue healed in the following 2 weeks and, on day 45, a 100% survival rate was achieved, Figure 16.

## 5. Conclusions

The combination of porphyrins or hydroporphyrins and micelles has led to the development of cancer nanotheranostic agents with enhanced photodynamic therapy effect and stronger fluorescence signal, resulting from increased specific photosensitizer accumulation which enables tumor tissue site-photoactivation. In some cases, the targeting effect was achieved by taking advantage of specific properties, such as pH-responsive or redox-responsive nanomicelles which ensure the delivery of the therapy and imaging agent at the same time at the tumor tissue. 

The nanomicellar systems allowed the incorporation of the different therapeutic agents (sensitizers for PDT, dyes or dots for PTT, DOX, or other chemotherapy agents) into the same structure, ensuring the same delivery system to the tumor and increasing the combination effects, resulting in enhanced antitumor efficacy when compared with each of the therapeutics individually assigned, in some cases, to synergistic effects. Recent advances in the development of theranostic systems included agents for potent imaging techniques namely, PET, MRI, or combined photoacoustic and fluorescence imaging which, jointly with combined therapies (PTT/PDT) or chemo- and PDT, led to a significant improvement of the precision of tumor localization and enhancement of efficacy of tumor treatment.
